# High Resolution Discrimination of Clinical *Mycobacterium tuberculosis* Complex Strains Based on Single Nucleotide Polymorphisms

**DOI:** 10.1371/journal.pone.0039855

**Published:** 2012-07-02

**Authors:** Susanne Homolka, Michaela Projahn, Silke Feuerriegel, Tanja Ubben, Roland Diel, Ulrich Nübel, Stefan Niemann

**Affiliations:** 1 Molecular Mycobacteriology, Research Center Borstel, Borstel, Germany; 2 Department of Pneumology, Medical School Hannover (MHH), Hannover, Germany; 3 Robert Koch Institute, Wernigerrode, Germany; University of Padova, Medical School, Italy

## Abstract

Recently, the diversity of the *Mycobacterium tuberculosis* complex (MTBC) population structure has been described in detail. Based on geographical separation and specific host pathogen co-evolution shaping MTBC virulence traits, at least 20 major lineages/genotypes have evolved finally leading to a clear influence of strain genetic background on transmissibility, clinical presentation/outcome, and resistance development. Therefore, high resolution genotyping for characterization of strains in larger studies is mandatory for understanding mechanisms of host-pathogen-interaction and to improve tuberculosis (TB) control. Single nucleotide polymorphisms (SNPs) represent the most reliable markers for lineage classification of clinical isolates due to the low levels of homoplasy, however their use is hampered either by low discriminatory power or by the need to analyze a large number of genes to achieve higher resolution. Therefore, we carried out *de novo* sequencing of 26 genes (approx. 20000 bp per strain) in a reference collection of MTBC strains including all major genotypes to define a highly discriminatory gene set. Overall, 161 polymorphisms were detected of which 59 are genotype-specific, while 13 define deeper branches such as the Euro-American lineage. Unbiased investigation of the most variable set of 11 genes in a population based strain collection (one year, city of Hamburg, Germany) confirmed the validity of SNP analysis as all strains were classified with high accuracy. Taken together, we defined a diagnostic algorithm which allows the identification of 17 MTBC phylogenetic lineages with high confidence for the first time by sequencing analysis of just five genes. In conclusion, the diagnostic algorithm developed in our study is likely to open the door for a low cost high resolution sequence/SNP based differentiation of the MTBC with a very high specificity. High throughput assays can be established which will be needed for large association studies that are mandatory for detailed investigation of host-pathogen-interaction during TB infection.

## Introduction

With one-third of the world’s population infected and approximately 2 million individuals dying of tuberculosis (TB) annually, bacteria of the *Mycobacterium tuberculosis* complex (MTBC) are among the most harmful pathogens [1]. The interaction of *M. tuberculosis* with HIV and the emergence of multidrug-resistant strains are further accelerating the TB epidemic [2]. Even more worrisome is that, in spite of nearly 100 years of intensive research, no vaccines are available to effectively protect adults from the development of pulmonary TB [3]. Thus, new approaches for understanding pathogenesis and disease dynamics are urgently needed.

The MTBC comprises seven closely related species with distinct host tropism: *M. tuberculosis*, *M. africanum* and *M. canettii* strains are obligate human pathogens, while *M. bovis* (bovine), *M. caprae* (goats), *M. microti* (rodents) and *M. pinnipedii* (seals) are considered as animal adapted organism [4]–[6]. Furthermore, recent studies demonstrate that these species can be subdivided in manifold phylogenetic lineages that show significant levels of functional genome variation and pathobiological characteristics [5]–[8].

First insights into the global population structure and the phylogenetic lineage composition have been gained by IS*6110* restriction fragment length polymorphism DNA fingerprinting (IS*6110* RFLP) and spacer oligonucleotide typing (spoligotyping) [9]–[11]. Following, large sequence and single nucleotide polymorphisms (LSP and SNP) have been successfully used to classify clinical isolates into main lineages with high specificity [6]–[8], [12].

However, due to the high similarity of MTBC strains on the genome level resolution was low when a practicable number of markers are analyzed [13]. More recently, multi locus variable number of tandem repeats (VNTR) analysis targeting 24 mycobacterial interspersed repetitive units (MIRU) has been established as a portable and discriminatory typing tool that allows simultaneously the investigation of strain transmission in epidemiological studies as well as the phylogenetic lineage classification of clinical isolates [14], [15].

However, when we compared the performance of spoligotyping, MIRU-VNTR-typing and sequence based (SNP) data for phylogenetic classification of a reference collection comprising major MTBC lineages, it turned out, that phylogenies inferred from spoligotyping or 15-loci-MIRU-VNTR were incongruent (mainly due to high levels of homoplasy), while phylogenetic trees derived from multilocus sequence data were highly congruent and statistically robust [16]. 24-loci-MIRU-VNTR led to higher resolution; however, it still was unable to detect all strain lineages with high statistical support [16]. These findings confirm that SNPs are more suited than MIRU-VNTR for defining deep phylogenetic groupings with very high confidence.

However, as outlined above, it is difficult to define a high discriminatory sequence based assay based on a limited number of targets due to the comparatively low sequence variability in clinical MTBC isolates [8], [17], [18]. Furthermore, the phylogenetic performance of SNP typing has mainly been investigated in reference strain collections [18]. An evaluation of specificity and sensitivity of SNP based lineage identification in population based epidemiological studies has not been carried out so far.

In this study, we accomplished *de novo* sequencing of 26 genes in 68 clinical isolates that represent all major phylogenetic lineages present in the MIRU-VNTR*plus* data base (www.MIRU-VNTRplus.org) [14], [19] in order to establish a sequence based assay with high discriminatory power. Sequence variations in the most variable 11 genes were evaluated in a population based study including strains obtained during one year of a longitudinal epidemiological survey carried out in Hamburg, Germany. Finally, based on these data, we designed a diagnostic algorithm including sequence analysis of just five genes that allow the classification of clinical isolates in 17 phylogenetic lineages in an easy, cost effective and highly specific way.

## Materials and Methods

### Bacterial Strains

Sequence analysis of 26 genes (Table 1) was carried out in 65 clinical isolates (Table 2) of the MIRU-VNTR*plus* reference collection [14], [19] as well as the ATCC reference strains H37Rv (ATCC 27294), *M. africanum* (ATCC 25420), and *M. bovis* (ATCC 19210). 21 different species/genotypes were represented by three clinical isolates (exception *M. pinipedii*: 2 strains). The targeted analysis of 11 genes was carried out in 104 strains collected during one year (2007) of an ongoing population based molecular epidemiological survey in Hamburg, Germany (Table S2).

**Table 1:** 26 Genes investigated.

Rv - number	Gene	whole gene sequence	Gene (bp)	PCR (bp)
Rv0129c	*fbpC*	yes	1023	1304
Rv0288	*esxH*	yes	291	788
Rv0388c	*ppe9*	yes	543	713
Rv0407	*fgd1*	yes	1011	1606
Rv0410c	*pknG*	yes	2253	2620
Rv0557	*mtfB*	yes	1137	1340
Rv1009	*rpfB*	yes	1089	1360
Rv1617	*pykA*	no	1419	315
Rv1811	*mgtC*	yes	705	880
Rv1884c	*rpfC*	yes	531	689
Rv1908c	*katG*	no	2223	1061
Rv1980c	*mpt64*	yes	687	858
Rv2032	*Acg*	yes	996	1359
Rv2389c	*rpfD*	yes	465	836
Rv2428	*ahpC*	no	588	237
Rv2430c	*ppe41*	yes	585	796
Rv2431c	*pe25*	yes	300	649
Rv2450c	*rpfE*	yes	519	938
Rv2609c	*–*	yes	1056	1314
Rv2610c	*pimA*	yes	1137	1302
Rv2611c	*–*	yes	951	1175
Rv2612c	*pgsA1*	yes	654	815
Rv2613c	*–*	yes	588	830
Rv2628	*–*	yes	363	521
Rv2629	*–*	yes	1125	1575
Rv3547	*–*	yes	456	846

PCR: polymerase chain reaction.

**Table 2:** Strains of the reference collection.

Sample Name	Species	Genotype
1449/02	*M. africanum*	West African 1a
1473/02	*M. africanum*	West African 1a
5434/02	*M. africanum*	West African 1a
10473/01	*M. africanum*	West African 1b
10494/01	*M. africanum*	West African 1b
1443/02	*M. africanum*	West African 1b
10514/01	*M. africanum*	West African 2
10517/01	*M. africanum*	West African 2
5468/02	*M. africanum*	West African 2
9550/00	*M. africanum*	West African 2 ATCC
4258/00	*M. bovis*	Bovis
751/01	*M. bovis*	Bovis
7540/01	*M. bovis*	Bovis
9564/00	*M. bovis*	Bovis ATCC
3040/99	*M. canettii*	Canettii
3041/99	*M. canettii*	Canettii
3151/08	*M. canetti*	Canettii
1694/00	*M. caprae*	Caprae
8986/99	*M. caprae*	Caprae
9577/99	*M. caprae*	Caprae
416/01	*M. microti*	Llama
8753/00	*M. microti*	Llama
1479/99	*M. microti*	Vole
7011/02	*M. pinipedii*	Seal
7739/01	*M. pinipedii*	Seal
12594/02	*M. tuberculosis*	Beijing
1500/03	*M. tuberculosis*	Beijing
1934/03	*M. tuberculosis*	Beijing
1417/02	*M. tuberculosis*	Cameroon
5390/02	*M. tuberculosis*	Cameroon
5400/02	*M. tuberculosis*	Cameroon
2637/02	*M. tuberculosis*	Delhi/CAS
7936/01	*M. tuberculosis*	Delhi/CAS
9915/01	*M. tuberculosis*	Delhi/CAS
1797/03	*M. tuberculosis*	EAI
4850/03	*M. tuberculosis*	EAI
947/01	*M. tuberculosis*	EAI
10469/01	*M. tuberculosis*	Ghana
10493/01	*M. tuberculosis*	Ghana
2570/02	*M. tuberculosis*	Ghana
9679/00	*M. tuberculosis*	H37Rv ATCC
2336/02	*M. tuberculosis*	Haarlem
4130/02	*M. tuberculosis*	Haarlem
9532/03	*M. tuberculosis*	Haarlem
7968/03	*M. tuberculosis*	LAM
8885/03	*M. tuberculosis*	LAM
946/03	*M. tuberculosis*	LAM
10459/03	*M. tuberculosis*	New-1
12591/02	*M. tuberculosis*	New-1
8870/03	*M. tuberculosis*	New-1
2151/03	*M. tuberculosis*	S-type
2318/06	*M. tuberculosis*	S-type
6411/05	*M. tuberculosis*	S-type
11313/03	*M. tuberculosis*	TUR
10264/03	*M. tuberculosis*	TUR
10529/03	*M. tuberculosis*	TUR
2169/99	*M. tuberculosis*	Uganda I
2201/99	*M. tuberculosis*	Uganda I
2333/99	*M. tuberculosis*	Uganda I
2176/99	*M. tuberculosis*	Uganda II
2191/99	*M. tuberculosis*	Uganda II
2253/99	*M. tuberculosis*	Uganda II
1657/03	*M. tuberculosis*	Ural
2679/03	*M. tuberculosis*	Ural
8431/03	*M. tuberculosis*	Ural
4412/04	*M. tuberculosis*	X-type
8431/05	*M. tuberculosis*	X-type
9953/04	*M. tuberculosis*	X-type

CAS: Central Asien; EAI: East African Indian; LAM: Latin American Mediterranean; TUR: Turkish.

All strains were analyzed by 24-loci-MIRU-VNTR and spoligotyping. Strains of the reference collection were further investigated by deletion analysis (region of difference; RD) to confirm phylogenetic classification (details at www.MIRU-VNTRplus.org).

### DNA Techniques

Extraction of DNA from mycobacteria was performed according to a standardized protocol [11]. All isolates were analyzed by spoligotyping [9] and 24 loci MIRU-VNTR as described previously [15]. The presence or absence of 16 RDs was analyzed by PCR using standard protocols available at the MIRU-VNTRplus webpage (www.MIRU-VNTRplus.org) [19].

For DNA sequence analysis 26 genes (Table 1) were amplified by polymerase chain reaction. Primer sequences are summarized in Table S1. Direct sequencing of PCR fragments was carried out using a commercially available sequencing kit (BigDye terminator v1.1, Applied Biosystems, Foster City, USA) and the ABI 3130XL sequencer according to the manufactures instructions (Applied Biosystems).

### Computer Analysis

Molecular typing data were analyzed with the BioNumerics software (version 6.5; Applied Maths, Sint-Martens-Latem, Belgium) as instructed by the manufacturer. Similarities of spoligotyping and MIRU-VNTR patterns were calculated by using the categorical coefficient. Classification of the Hamburg strain collection in MTBC genotypes was carried out by using the MIRU-VNTR*plus* database [14], [19]. Analysis of sequence data and SNP detection was performed by using SeqScape v2.6 software (Applied Biosystems). Genome sequences of *M. tuberculosis* H37Rv (http://tuberculist.epfl.ch/) were used as a reference sequence. Calculation of a maximum parsimony phylogenetic tree based on SNP data of the Hamburg strain collection was performed with BioNumerics. A maximum likelihood phylogenetic tree was constructed based on an alignment of SNPs discovered in 26 genes from the reference collection isolates by applying Treefinder software (available at http://www.treefinder.de/) and using the HKY model of DNA substitution. Bootstrap support was calculated based on 1,000 replicates.

More details about procedures for analysis of typing data are described elsewhere [19]–[21].

## Results

In this study we investigated the performance of sequence based analysis for highly discriminatory phylogenetic classification of clinical MTBC isolates using *de novo* sequencing of 26 genes or part of the genes in a reference collection of 68 pre-selected MTBC strains which represent all major phylogenetic lineages (three strains for most of the lineages, Table 2). This collection included 54 strains of human-adapted (*M. tuberculosis, M. africanum* and *M. canettii*) as well as 11 strains of animal adapted lineages (*M. bovis, M. microti, M. pinnipedii, M. caprae*) and the ATCC strains H37Rv, *M. bovis* and *M. africanum*. All strains were previously classified into phylogenetic lineages based on spoligotyping, MIRU-VNTR and deletion typing [19].

The analyzed genes comprised cell wall associated genes, antigens, genes involved in metabolism, resuscitation factors and other genes of special interest e.g. those that have been described to have an overall higher mutation rate [7].

In total, we determined DNA sequences (exception: strain 8885/03 no sequence data for Rv2431c) of 20078 base pairs (bp) for each strain which correspond to ∼0.46% of the MTBC genome. All sequences determined were compared to the H37Rv reference genome to identify possible variations e.g. SNPs, deletions or insertions. In total we found 161 sequence variations resulting in an average mutation density of 1.18×10^−4^/bp. As described before, sequence variation was variable among the genes investigated (Table S3). For example, antigens (Rv0288, Rv1980c) are more conserved than cell wall associated genes (Rv0577).

Six of the mutations detected were deletions (n = 5) or insertions (n = 1, Table S5). In accordance with previous findings [7], [16], the majority of the 155 SNPs detected were non-synonymous mutations (n = 89, 57.42%, Table S3), while only 66 of them were synonymous (42.58%).

Besides, 66 of all 155 SNPs detected were only found in the three *M. canettii* strains investigated. Thereof a significantly higher SNP density of 1.10×10^−3^/bp could be calculated for *M. canettii* (or *M. prototuberculosis*) strains confirming that these strains are considered to have a longer evolutionary history. Accordingly, the ratio of non-synonymous to synonymous polymorphisms was much lower in comparison to other MTBC strains (25 (37.88%) vs. 41 (62.12%)).

**Figure 1:**
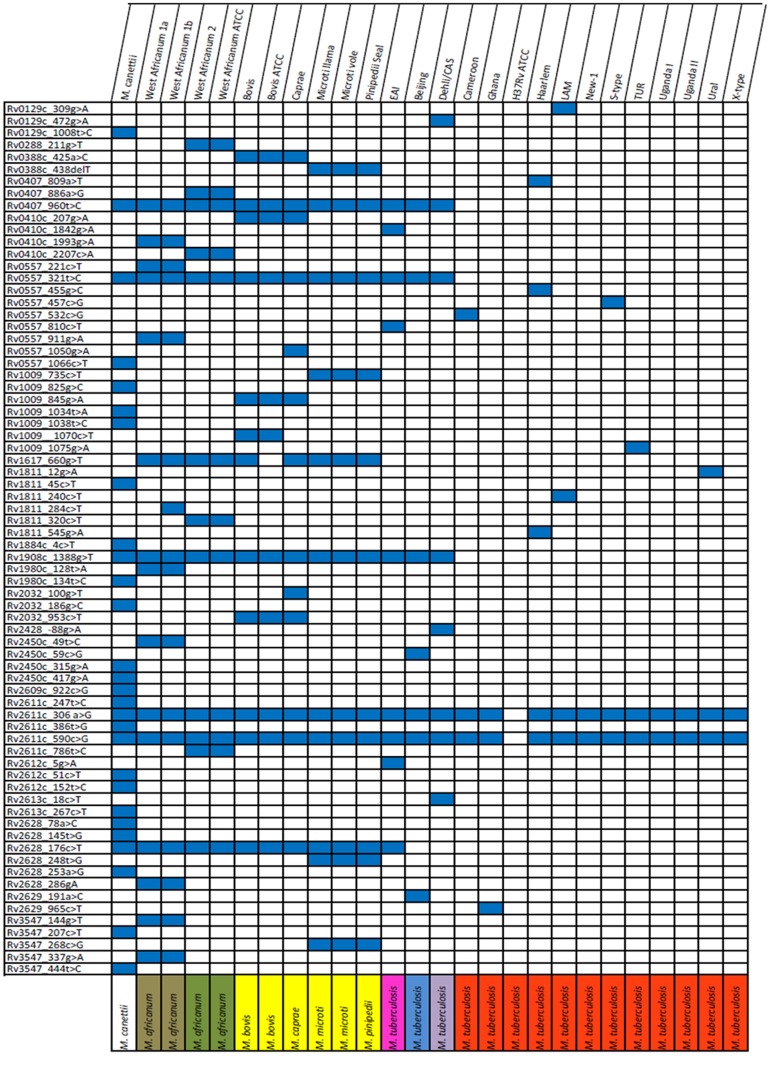
Phylogenetic informative sequence variations. CAS: Central Asien; EAI: East African Indian; LAM: Latin American Mediterranean; TUR: Turkish.

**Figure 2:**
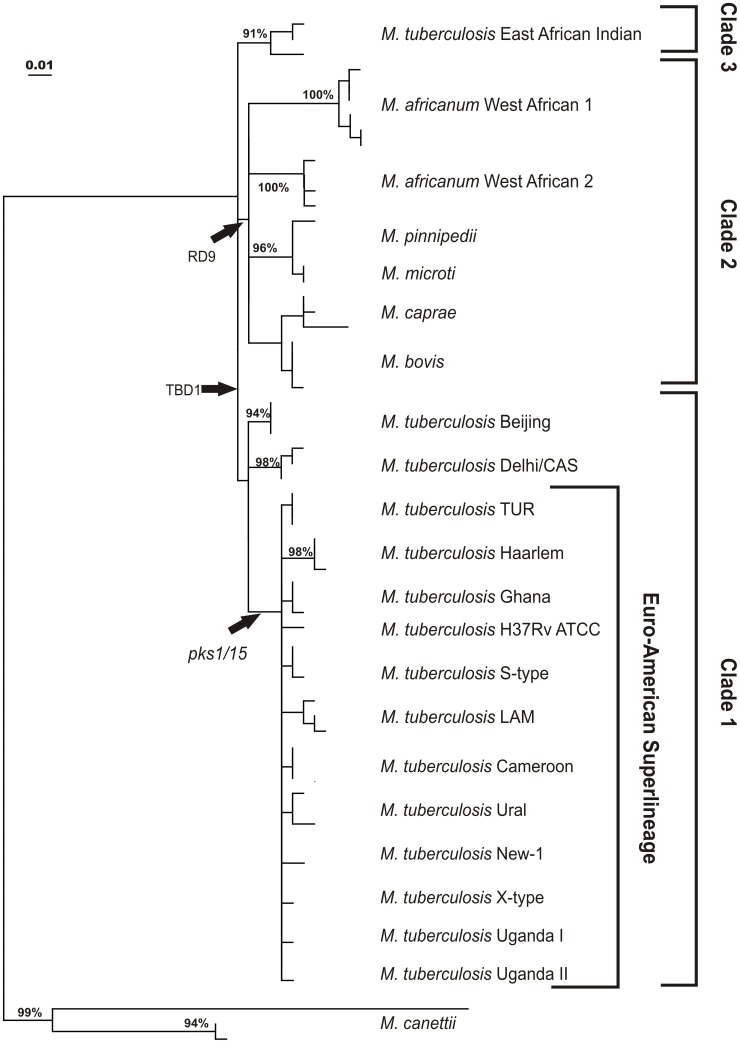
Maximum Likelihood Tree based on sequence data from a reference collection. Bootstrap support >90% is indicated. CAS: Central Asien; LAM: Latin American Mediterranean; TUR: Turkish.

As we correlate the occurrence of SNPs with the phylogenetic strain classification, it appeared that out of the 161 polymorphisms detected 59 SNPs are genotype-specific meaning that they were only found in MTBC strains of one specific phylogenetic sub-lineage e.g. strains of the Haarlem genotype or the Beijing genotype (Figure 1, *M. africanum* West African 1a and 1b are considered as one genotype in this context). An additional number of 12 SNPs were found to be specific for deeper branches in the phylogeny defining larger groups such as the Euro-American lineage or so called clade 2 strains (Figure 1 and 2). One of the detected deletions (Rv0388c_438delT) was specific for all *M. bovis* and *M. caprae* strains investigated (Figure 1). In addition, all phylogenetic informative SNPs correlate with the occurrence of particular deletions e.g. the pks1/15 deletion that is specific for the Euro-American lineage (Figure 2).

Overall, when using the genotype-specific SNPs, we were able to discriminate nearly all lineages except the very closely related *M. tuberculosis* genotypes New-1, Uganda and X-type as well as *M. microti* and *M. pinnipedii* strains. Again, due to the much higher number of polymorphisms, *M. canettii* strains showed the most genotype specific variability (22/59) followed by *M. africanum* West African 1 (9/59) and West African 2 isolates (5/59) (Figure 1). To further investigate the phylogenetic informative content of the generated data and the population structure of the strains investigated, we calculated a maximum likelihood tree on the basis of the polymorphisms detected (Figure 2). This sequence based tree groups the analyzed MTBC isolates into the most common phylogenetic lineages defined by MIRU-VNTR typing and spoligotyping. The MTBC strains fall into three clearly distinct major groups: clade 1 comprising the classical *M. tuberculosis* strains, clade 2 comprising *M. africanum* and the animal isolates, and clade 3 comprising the *M. tuberculosis* EAI strains that are clearly separated from all other *M. tuberculosis* strains investigated.

In comparison to previous studies, our analyses confirm that sequence data are entirely suitable for the phylogenetic classification of MTBC strains when well characterized strain collections are analyzed [16]. However to further evaluate this notion in a more unbiased manner we analyzed the most variable 11 of 26 genes in a population based collection of 104 MTBC strains obtained in the year 2007 from tuberculosis patients living in Hamburg, Germany by *de novo* sequencing. As a first step, all strains were classified into phylogenetic lineages based on spoligotyping (Table S2). This revealed a highly diverse population structure comprising strains of several phylogenetic lineages ranging from *M. africanum* West African 1, *M. bovis*, to *M. tuberculosis* EAI, Beijing, Delhi/CAS, Haarlem, Cameroon, Ghana, LAM, S-type, TUR and Uganda strains. However, a larger group of strains could not be allocated to a particular lineage and was confirmed to belong to the Euro-American lineage based on deletion typing of pks1/15 (data not shown).

The sequence data revealed the presence of 61 SNPs of which 41 were non-synonymous and 20 synonymous polymorphisms (Table S4). In addition, 10 of the strains investigated had a 70 bp deletion and three a 102 bp deletion (specific for the TUR genotype) in Rv2450c (Table S6). Overall, 29 out of 61 SNPs determined were SNPs previously found to be either genotype or lineage specific in the reference collection. Again, the occurrence of these SNPs was in nearly complete concordance with phylogenetic strain classification based on MIRU-VNTR and spoligotyping data. Only one strain was classified as “not defined” based on spoligotyping data (as no specific spoligotyping signature was present) but had a SNP specific for the TUR lineage (Table S2; 8918/07, Rv1009_1075g>A). Cluster analysis based on 24-loci MIRU-VNTR typing data grouped this strain together with the other three TUR strains investigated, thus, confirming the SNP typing result (data not shown).

Interestingly, the sequence data also allowed the further sub-classification of three strains of the EAI genotype into the EAI “Manila” sub-type [22] based on two SNPs (Rv0410c_2117t>C, Rv1009_724g>A). In addition, the generated *de novo* sequence data revealed a new SNP in Rv2628 (4t>C) that is specific for 18 strains of the Hamburg collection which were previously classified into the Euro-American lineage, thus, most likely defining a new genotype (Hamburg lineage). When spoligotyping data were considered, these strains showed no clear criteria for close relationship, while MIRU-VNTR typing grouped them together (data not shown) confirming a clonal relationship. Finally, all strains with the 70 bp deletion in Rv2450c belong to the Hamburg lineage only indicating a further sub-branching in this new described lineage.

**Figure 3:**
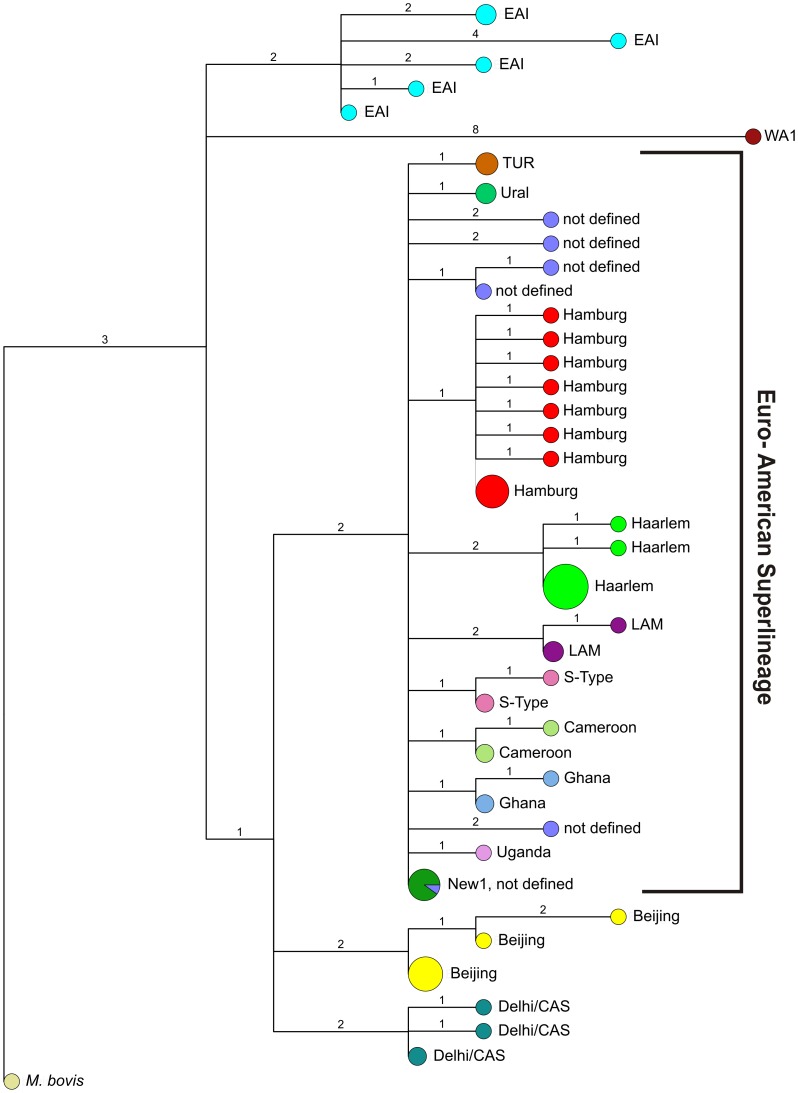
Maximum Parsimomy Tree based on sequencing analyses data of 11 genes in a population based strain collection from Hamburg, Germany. WA1, *M. africanum* West African 1; CAS, *M. tuberculosis* Central Asien; EAI, *M. tuberculosis* East African Indian; LAM, *M. tuberculosis* Latin American Mediterranean; TUR, *M. tuberculosis* Turkish.

The superior classification of the strains into various phylogenetic lineages is also shown in the maximum parsimony tree calculated based on the SNP data (Figure 3). The tree reveals the high resolution classification of the analyzed strains into the phylogenetic lineages of the MTBC, obviously with high specificity and a high sensitivity as it is shown by the clear identification of the single *M. africanum* West African 1 and *M. bovis* strain in the study population, respectively. Again SNP based phylogeny reflects the phylogenetic classification of clinical isolates based on classical typing methods e.g. MIRU-VNTR typing (data not shown), however, with a much higher accuracy/confidence for the classification of known and new groups esp. in the Euro-American lineage.

**Figure 4:**
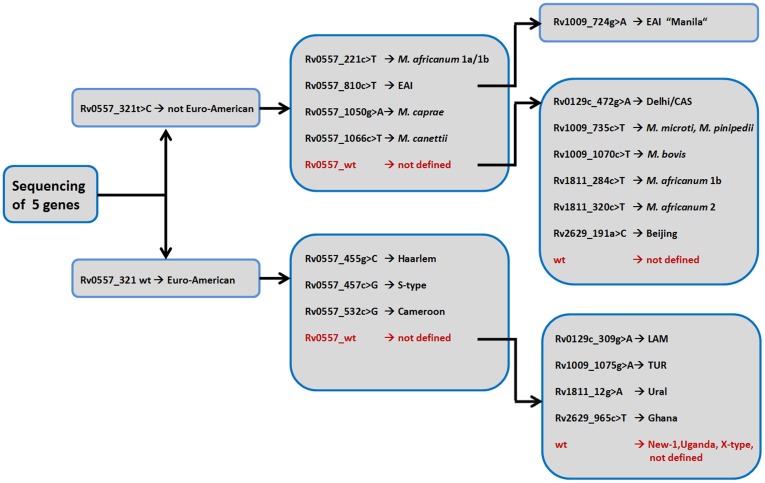
Sequence based algorithm for the classification of clinical isolates in 17 different genotypes and phylogenetic main groups. *M. africanum* 1a/1b, West African 1a and West African 1b; *M. africanum* 2, West African 2; CAS, *M. tuberculosis* Central Asien; EAI, *M. tuberculosis* East African Indian; LAM, *M. tuberculosis* Latin American Mediterranean; TUR, *M. tuberculosis* Turkish; wt: wildtype.

Finally, as the analysis of 26 or even 11 genes for larger clinical isolates is still time consuming and costly, we developed a decision tree comprising the most variable genes but still allowing the classification of MTBC strains into the most common phylogenetic lineages. This diagnostic algorithm comprises sequence analysis of five genes only (Rv0557, Rv0129c, Rv1009, Rv1811 and Rv2628) and enables the specific and high resolution classification of clinical MTBC isolates into 17 different genotypes or phylogenetic lineages as well as the definition of the *M. tuberculosis* EAI “Manila” sub-group (Figure 4). This is likely to open the door for cost efficient and highly specific sequence or SNP based phylogenetic classification of clinical isolates in larger epidemiological studies.

## Discussion

Genetic variability of clinical isolates on the strain level resulting in various phylogenetic lineages with potential pathobiological differences as well as the global population structure of MTBC strains were neglected for decades. Initial studies demonstrated a high similarity on the DNA level (a sequence similarity of 99.95%). Accordingly, members of the MTBC are considered as genetically highly monomorph bacteria [6], [18], [23].

However, recent studies showed that clinical isolates are more genetically distinct than previously assumed and that the genetic background of strains is responsible for variable pathobiological characteristics [7], [24], [25]. Only recently, it was shown that genomic variation is mainly driven by a high degree of diversity in form of SNPs, the majority of them being non-synonymous with potential functional consequences [7], [16].

The pathobiological importance of this genotype diversity has been confirmed by demonstrating enhanced spread of strains of particular lineages, e.g., in the context of multidrug resistance [26], defining lineage-specific disease characteristics [27], [28], and confirming host–pathogen co-evolution and specific host–pathogen interactions [29]–[31]. Very recently, we demonstrated significant levels of phylogenetically based transcriptome diversity of clinical MTBC isolates upon infection of mouse macrophages using microarrays [24].

These preliminary findings are challenging and argue strongly for larger research projects investigating MTBC pathobiological diversity in the context of TB control or drug and vaccine development. However, to study the importance of MTBC pathogenetic diversity and define local and global population structure, a valid identification of various phylogenetic lineages is a precondition. Ideally, the applied method shall be cost effective and applicable in a high throughput format in larger studies. Recently we showed that SNPs are likely to be the most valid markers due to the very low level of homoplasy and that they are ideally suited for defining phylogenetic groupings with very high confidence [25]. Additionally, recent genome analysis revealed the presence of high numbers of lineage specific SNPs that allow a highly robust phylogeny [16]. However, due to the comparably low level of diversity (1 SNP in 3000 bp) in the genomes of MTBC strains, it is still challenging to define a SNP panel comprising a smaller number of genes that allow a cost effective discrimination of the MTBC in a high or low throughput manner in laboratories not equipped with high throughput sequencing technologies.

In the present study, we investigated the genetic diversity of clinical isolates representing different genotypes in 26 genes by *de novo* sequencing generating approx. 20.000 bp sequence information per strain. The gene set comprised cell wall associated genes, antigens, genes involved in metabolism, resusciation factors and other genes of special interest [7]. In addition, as an important step towards a most valid SNP classification scheme, we carried out the first unbiased validation of the SNPs determined in the most variable set of 11 genes in a population based strain collection covering all strains from cases with pulmonary TB from the city of Hamburg, Germany. Finally, we were able to define a diagnostic algorithm based on the analysis of just five genes which identify the majority of MTBC phylogenetic lineages with high confidence at minimal costs.

In comparison to other SNP based classification schemes, our assay is clearly superior in terms of the small number of genes/SNPs to be analyzed as well as in the number of genotypes that can be distinguished. So far, Filliol et al. described in 2006 a minimal set of 45 informative SNPs to distinguish MTBC strains [12]. More recently, few studies described SNP sets in cell wall biosynthesis-associated genes [32], in genes involved in replication, repair and recombination (3R) [33], as well as an extended genome wide set of 35 SNPs [34]. However, all studies are hampered by limited resolution and none of these provided an in depth evaluation in an unbiased population based strain collection reflecting the global diversity. On the contrary, our *de novo* sequencing approach revealed a high number of lineage specific SNPs (several of which have not been described before), that finally define specific markers for nearly all lineages included in a fairly low number of genes to be analyzed.

Our data on lineage specific SNPs are in accordance with previous data generated by *de novo* sequencing of particular gene sets [7] and by whole genome re-sequencing of MTBC reference strains [25], [32], thus further affirming the validity of the SNPs included as markers for particular lineages. This has been further confirmed by the fact that the SNP based classification in both study collections investigated was in full concordance with previous classification based on classical typing techniques. Taken together, our data confirm that SNPs show a very low level of homoplasy and are eminently suitable as distinct phylogenetic markers. Therefore, the described diagnostic algorithm based on sequencing of five highly variable genes maybe a new, easy and cost effective method for the valid identification of MTBC strains in epidemiological studies and complements our diagnostic gold standard genotyping techniques like spoligotyping and MIRU-VNTR typing.

The sequence data generated in this study also provided in depth insights into the population structure and evolution of the MTBC. The phylogeny derived from the SNP data obtained, confirm the population structure described by classical molecular typing methods and by a previous large scale multi locus sequencing approach analyzing a different set of genes in a slightly divergent global collection of clinical isolates [4], [5]. The phylogenetic tree confirms the presence of at least 17 MTBC lineages in the reference collection that are all confirmed by the presence of specific SNPs. Furthermore, our data sustain the existence of major clades, clade 1 comprising various *M. tuberculosis* genotypes ranging from Beijing, Delhi/CAS to Haarlem and TUR and clade 2 strains comprising *M. africanum* West African 1 and 2 and various animal pathogenic species such as *M. bovis* and *M. caprae*. This is in accordance with previous studies based on 24 loci MIRU-VNTR typing and sequence based analysis [4], [7]. However, our data reveal the presence of a potential additional major MTBC clade as *M. tuberculosis* EAI strains are clearly separated from all other *M. tuberculosis* strains, thus likely representing a third independent main lineage.

Additionally, the present study provides a deeper branching order of several lineages particularly within clade 2 that can be investigated by sequential occurrence of shared SNPs. This analysis nicely confirms the very close relationship of *M. bovis* and *M. caprae*, *M. pinnipedii* and *M. microti,* and both *M. africanum* lineages. It also indicates that *M. tuberculosis* EAI strains might be more closely related to clade 2 rather than to clade 1 strains, which also raises the question if all MTBC strains are derived from a single common ancestor.

Another debatable point is whether *M. africanum* lineages really constitute a common species as West African 1 and West African 2 strains are separated by several SNPs rendering to a genetic distance larger than e.g. between *M. bovis* and *M. caprae* strains. Our finding is in concordance with the analysis of the presence or absence of large chromosomal deletions and with whole genome analysis of few strains also clearly separating both *M. africanum* lineages [25]. Accordingly, species naming and classification needs to be revised in the light of new genome based data. Furthermore, the variability of clade 2 lineages might be rather underestimated as the majority of studies (including ours) are mainly focused on classical *M. tuberculosis* strains and include only few strains of *M. africanum* or the animal pathogenic species. The same accounts for the population structure and genetic variability of *M. tuberculosis* EAI strains that might constitute a largely uninvestigated new major branch within the MTBC.

The *de novo* sequencing of 11 genes in the population based collection of MTBC strains from Hamburg revealed further weaknesses of previous studies investigating MTBC population structure by genomic means that have been mainly based on reference collections of known genotypes. In addition to the high concordance of SNP data with previous strain classification in known lineages, we discovered a potential new subgroup within the Euro-American lineage. Overall, 18 of 33 Euro-American strains of the Hamburg collection that were not classified in a particular lineage by spoligotyping or MIRU-VNTR data carried a sequence variation in Rv2628 (4t>C) and could be defined as a new Euro-American sublineage (Hamburg lineage). Beyond that, 10 of these 18 strains showed a 70 bp deletion in Rv2450c which identified another subgroup of the Hamburg lineage. These data indicate that the diversity and the population structure of the Euro- American lineage might be largely underestimated and is not well defined yet. Similar studies applying *de novo* sequence analysis either based on selected genes or based on whole genomes are necessary to better describe the phylogenetic diversity of the MTBC, esp. of those strains which are not classified yet. Besides, larger studies on the diversity of clade 2 and the new defined clade 3 strains are urgently needed.

In accordance with previous studies we also observed that the overall mutation rates are varying between the different genes in dependence of the function of the gene product which was published for other organism previously [23]. For example, antigens coded by Rv0288 and Rv1980c are comparatively less variable than cell wall associated genes like Rv0557 which confirm our recent data obtained from whole genome sequencing of a smaller collection of MTBC strains [35] indicating that antigens of *M. tuberculosis* are evolutionary hyperconserved and nearly invariable. Contrastingly, functional sequence variations in cell wall associated genes like in Rv0557 may lead to a higher fitness of strains or a selection advantage similar to mutations described for the *embB* gene coded for a transmembrane arabinosyltransferase mediating ethambutol resistance [36], [37].

Out of all mutations detected in the reference collection 89 let to an exchange in the amino acid sequence whereas 66 SNPs were synonymous. That implies a non-synonymous to synonymous ratio of 1.35 which correlates with the described data for *M. bovis* and CDC1551 in comparison to the H37Rv reference strain [18], [38]. Our data confirm the hypothesis of a reduced purifying selection in clinical MTBC isolates [7] caused by the small effective population size and the repeated bottleneck events during transmission.

In conclusion, the diagnostic algorithm developed in our study is likely to open the door for a high resolution sequence/SNP base differentiation of the MTBC with a very high specificity. Due to the small number of genes investigated, low cost sequencing assays or Real Time PCR assays are desirable. Furthermore, our in depth *de novo* sequencing data provide a detailed phylogenetic scenario for the MTBC that is in major concordance with previous studies. However, both, the data derived from the global reference collection as well as from the population based strain collection indicate that the population diversity in several branches is largely underestimated and lineage/species naming requires revision taken new genome data into account. Thus, our data warrant further in depth analysis of the population structure of the MTBC esp. of *M. africanum* and *M. tuberculosis* EAI strains. In addition, future studies should step back from reference strain collections and include more unbiased collections e.g. from population based epidemiological investigations.

## Supporting Information

Table S1 **Primer sequences.**
(DOCX)

Table S2 **Strain collection from Hamburg in the year 2007.**
(DOCX)

Table S3 **SNPs detected in the reference collection.**
(DOCX)

Table S4 **SNPs detected in Hamburg collection 2007.**
(DOCX)

Table S5 **Additional variations in strains of the reference collection.**
(DOCX)

Table S6 **Additional variations in strains from Hamburg, Germany.**
(DOCX)
