# Supplementary material for: High Resolution Discrimination of Clinical Mycobacterium tuberculosis Complex Strains Based on Single Nucleotide Polymorphisms
Source: PLoS One. 2012 Jul 2;7(7):e39855. doi: 10.1371/journal.pone.0039855 (PMC3388094; doi:10.1371/journal.pone.0039855)
Supplement: Table S1 — Primer sequences. (DOCX) [file pone.0039855.s001.docx]

**Table S1. Primer sequences.**

| Primer | Sequence | Position |
| --- | --- | --- |
| Rv0129c - F | 5`- GAA CCT CCA CGC CCG CAA C -3` | - 175 |
| Rv0129c - F | 5`- GCG CTG CGG CCA CGA CAT TC-3` | + 1129 |
| Rv0288 - F^1^ | 5`- GAG AGG GGG AGG CGA CGG CTA CC -3` | - 411 |
| Rv0288 - R^1^ | 5`- TCC CCG CCC CAA TGG TTT CAG C -3` | + 356 |
| Rv0388c - F | 5`- CCC AGC CAC GCA GCG CTG AG -3` | - 49 |
| Rv0388c - R | 5`- CTC GAT GGG GTT CGG AAT G -3` | + 646 |
| Rv0407 - F^1^ | 5`- CGT GGC CGC GAG CGA GGT GAA -3` | - 189 |
| Rv0407 - R^1^ | 5`- CGC CCG AAC CGT CAA CAA CAC TGG -3` | + 1394 |
| Rv0407 - S1^1^ | 5`- CGG AGT TCA AGG AGC GGT TCG -3` | + 368 |
| Rv0410c - F^1^ | 5`- CTG GCC GGG CTG GTA GAG GAA GAC -3` | - 227 |
| Rv0410c - R^1^ | 5`- CCG ATC GCC GTG CTG GTG GTT -3` | + 2373 |
| Rv0410c - S1^1^ | 5`- TGA GGC CCT GAT GAC CAA CCC -3` | + 270 |
| Rv0410c - S2^1^ | 5`- GGC GCT GAG CTA CCT GCA TTC -3` | + 786 |
| Rv0410c - S3^1^ | 5`- GCG CAC ACC GAC GTG TAT CTG -3` | + 1285 |
| Rv0557 - F | 5`- CTG GAC AAG CGG TTG GAA C -3` | - 112 |
| Rv0557 - R | 5`- GTC ATA CTT GCG GGC GAC G -3` | + 1210 |
| Rv1009 - F^1^ | 5`- GGC CCA TTT TGC TTT TTG TT -3` | - 105 |
| Rv1009 - R^1^ | 5`- GGC CCG ACC TCC AAA ACC AG -3` | + 1236 |
| Rv1617 - F^2^ | 5`- CGT TGC CCG GAA TGA ACG TG -3` | + 473 |
| Rv1617 - R^2^ | 5`- GGA TGG CTC GCT TCT GTA CC -3` | + 768 |
| Rv1811 - F | 5`- CGC CTA GGC TCA AAC TGC TG -3` | - 78 |
| Rv1811 - R | 5`- CAA TAC CCG GCG GAT CTA CC -3` | + 783 |
| Rv1884c - F^1^ | 5`- CAA CGG GCC CTG ACG ACA -3` | - 68 |
| Rv1884c - R^1^ | 5`- GCG CTG CCA TGT GAA CTG TG -3` | + 602 |
| Rv1908c - F | 5`- GAA ACA GCG GCG CTG ATC GT -3` | + 781 |
| Rv1908c - R | 5`- GCCTTGTCGAGCAGCATGTAC -3` | +1841 |
| Rv1980c - F^1^ | 5`- CAG CGC GAT GCC CTA TGT -3` | - 102 |
| Rv1980c - R^1^ | 5`- TGG GCG CAC CGA ACA CTC -3` | + 739 |
| Rv2032 - F^1^ | 5`- TGG CGC TGA ACG GGA AGG GTG GT -3` | - 225 |
| Rv2032 - R^1^ | 5`- CTC AGC CGG CCG GGG ACG AAT AGC -3` | + 1111 |
| Rv2032 - S1^1^ | 5`- GCC GGA TTG GGA CTT GGT -3` | + 390 |
| Rv2389c - F^1^ | 5`- TGG CGG CGT CGG TCA AAT CAG C -3` | - 169 |
| Rv2389c - R^1^ | 5`- CCA GCT GGG CCG GGG TGT AGG TA -3` | + 645 |
| Rv2428 - F | 5`- ACC ACT GCT TTG CCG CCA CC -3` | - 178 |
| Rv2428 - R | 5`- CCG ATG AGA GCG GTG AGC TG -3` | + 40 |
| Rv2430c - F | 5`- CGG CGG CGG TGG TTC TTG AG -3` | - 144 |
| Rv2430c - R | 5`- TGA AAG CCG CAG CAA TCG TT -3` | + 633 |
| Rv2431c - F | 5`- ATC GTC GGG TGG CGT TGT GT -3` | - 197 |
| Rv2431c - R | 5`- CAT CCA ACG ACC TCC ACG CC -3` | + 433 |
| Rv2450c - F^1^ | 5`- CGG GCA GCA AGA AAT CTC AAC AAT -3` | - 240 |
| Rv2450c - R^1^ | 5`- GCC CGG GCC AGG TAT TC -3` | + 681 |
| Rv2609c - F | 5`- TGG CCG ATG GAC TGA TTG C -3` | - 169 |
| Rv2609c - R | 5`- TCA ACG ACG CCC TGT CCG GA -3` | + 1126 |
| Rv2609c - S1 | 5`- GGC CCG CCG CTT CCA CAA C -3` | + 366 |
| Rv2610c - F | 5`- CAG AAC ATT GCC GCC CAT C -3` | - 109 |
| Rv2610c - R | 5`- GCC GAA CGC AAC CAG CAC A -3` | + 1175 |
| Rv2610c - S1 | 5`- CCG ATT GTG GCG ACA TTT CA -3` | + 334 |
| Rv2611c - F | 5`- GGC GTT GTC GGT GGG GAT GT -3` | - 126 |
| Rv2611c - R | 5`- TGC GCA TCA CCT CGG CAA GC -3` | + 1030 |
| Rv2611c - S1 | 5`- CCG GCT GCC GAC GAT AAA C -3` | + 291 |
| Rv2612c - F | 5`- ACC ATC ATC GGG GGC TCC A -3` | - 83 |
| Rv2612c - R | 5`- CAT ACG CCC AGT CGG TCG C -3` | + 714 |
| Rv2613c - F | 5`- TCA TGG TGT TGG CGG GTG AT -3` | - 174 |
| Rv2613c - R | 5`- CAG CAG TCC CCT GGC GAT C -3` | + 638 |
| Rv2628 - F^1^ | 5`- GGC GCG ACC GGG CAC ATC -3` | - 83 |
| Rv2628 - R^1^ | 5`- GCG GGA AGG CAT AGG GAC CAA AGT -3` | + 415 |
| Rv2629 - F | 5`- CCG CTG GAC CGC ACA ACG AT -3` | - 275 |
| Rv2629 - R | 5`- GAT TCG CAA GTC CGC CGT AT -3` | + 1281 |
| Rv3547- F^1^ | 5`- CGA GCG CAC CGA CCA GAG C -3` | - 196 |
| Rv3547- R^1^ | 5`- GCA TGG CCC GCA GGT GGA CAA -3` | + 630 |

Hershberg et al.^1^ Keating et al. ^2^; – position before start codon; + position after start codon

^1^Hershberg R, Lipatov M, Small PM, Sheffer H, Niemann S, et al. (2008) High functional diversity in Mycobacterium tuberculosis driven by genetic drift and human demography. PLoS Biol 6: e311. doi:10.1371/journal.pbio.0060311.

^2^Keating LA, Wheeler PR, Mansoor H, Inwald JK, Dale J, et al. (2005) The pyruvate requirement of some members of the Mycobacterium tuberculosis complex is due to an inactive pyruvate kinase: implications for in vivo growth. Molecular Microbiology 56: 163–174. doi:10.1111/j.1365-2958.2005.04524.x.
