# Supplementary material for: High Resolution Discrimination of Clinical Mycobacterium tuberculosis Complex Strains Based on Single Nucleotide Polymorphisms
Source: PLoS One. 2012 Jul 2;7(7):e39855. doi: 10.1371/journal.pone.0039855 (PMC3388094; doi:10.1371/journal.pone.0039855)
Supplement: Table S2 — Strain collection from Hamburg in the year 2007. (DOCX) [file pone.0039855.s002.docx]

**Table S2. Strain collection from Hamburg in the year 2007.**

| Sample Name | Species | Genotype Spoligotyping | Genotype SNP typing |
| --- | --- | --- | --- |
| 6629/07 | *M. africanum* | West African 1 | West African 1 |
| 1110/07 | *M. bovis* | Bovis | Bovis |
| 10468/07 | *M. tuberculosis* | Beijing | Beijing |
| 1047/07 | *M. tuberculosis* | Beijing | Beijing |
| 11156/07 | *M. tuberculosis* | Beijing | Beijing |
| 1339/07 | *M. tuberculosis* | Beijing | Beijing |
| 1465/07 | *M. tuberculosis* | Beijing | Beijing |
| 2072/07 | *M. tuberculosis* | Beijing | Beijing |
| 2280/07 | *M. tuberculosis* | Beijing | Beijing |
| 3431/07 | *M. tuberculosis* | Beijing | Beijing |
| 4484/07 | *M. tuberculosis* | Beijing | Beijing |
| 4825/07 | *M. tuberculosis* | Beijing | Beijing |
| 5250/07 | *M. tuberculosis* | Beijing | Beijing |
| 8088/07 | *M. tuberculosis* | Beijing | Beijing |
| 8230/07 | *M. tuberculosis* | Beijing | Beijing |
| 9589/07 | *M. tuberculosis* | Beijing | Beijing |
| 2111/07 | *M. tuberculosis* | Cameroon | Cameroon |
| 3995/07 | *M. tuberculosis* | Cameroon | Cameroon |
| 6668/07 | *M. tuberculosis* | Cameroon | Cameroon |
| 11584/07 | *M. tuberculosis* | Delhi/CAS | Delhi/CAS |
| 3064/07 | *M. tuberculosis* | Delhi/CAS | Delhi/CAS |
| 3987/07 | *M. tuberculosis* | Delhi/CAS | Delhi/CAS |
| 5357/07 | *M. tuberculosis* | Delhi/CAS | Delhi/CAS |
| 11135/07 | *M. tuberculosis* | EAI | EAI |
| 6656/07 | *M. tuberculosis* | EAI | EAI |
| 6667/07 | *M. tuberculosis* | EAI | EAI |
| 6942/07 | *M. tuberculosis* | EAI | EAI |
| 7828/07 | *M. tuberculosis* | EAI | EAI |
| 8224/07 | *M. tuberculosis* | EAI | EAI |
| 9300/07 | *M. tuberculosis* | EAI | EAI |
| 4524/07 | *M. tuberculosis* | Ghana | Ghana |
| 6456/07 | *M. tuberculosis* | Ghana | Ghana |
| 7333/07 | *M. tuberculosis* | Ghana | Ghana |
| 10471/07 | *M. tuberculosis* | Haarlem | Haarlem |
| 11586/07 | *M. tuberculosis* | Haarlem | Haarlem |
| 1379/07 | *M. tuberculosis* | Haarlem | Haarlem |
| 1801/07 | *M. tuberculosis* | Haarlem | Haarlem |
| 2463/07 | *M. tuberculosis* | Haarlem | Haarlem |
| 2466/07 | *M. tuberculosis* | Haarlem | Haarlem |
| 3087/07 | *M. tuberculosis* | Haarlem | Haarlem |
| 3484/07 | *M. tuberculosis* | Haarlem | Haarlem |
| 3986/07 | *M. tuberculosis* | Haarlem | Haarlem |
| 4132/07 | *M. tuberculosis* | Haarlem | Haarlem |
| 4794/07 | *M. tuberculosis* | Haarlem | Haarlem |
| 6577/07 | *M. tuberculosis* | Haarlem | Haarlem |
| 6699/07 | *M. tuberculosis* | Haarlem | Haarlem |
| 6900/07 | *M. tuberculosis* | Haarlem | Haarlem |
| 7952/07 | *M. tuberculosis* | Haarlem | Haarlem |
| 8681/07 | *M. tuberculosis* | Haarlem | Haarlem |
| 8917/07 | *M. tuberculosis* | Haarlem | Haarlem |
| 8941/07 | *M. tuberculosis* | Haarlem | Haarlem |
| 9297/07 | *M. tuberculosis* | Haarlem | Haarlem |
| 9524/07 | *M. tuberculosis* | Haarlem | Haarlem |
| 9679/07 | *M. tuberculosis* | Haarlem | Haarlem |
| 9798/07 | *M. tuberculosis* | Haarlem | Haarlem |
| 9952/07 | *M. tuberculosis* | Haarlem | Haarlem |
| 3258/07 | *M. tuberculosis* | LAM | LAM |
| 5599/07 | *M. tuberculosis* | LAM | LAM |
| 852/07 | *M. tuberculosis* | LAM | LAM |
| 9382/07 | *M. tuberculosis* | LAM | LAM |
| 9893/07 | *M. tuberculosis* | not defined | Hamburg |
| 10583/07 | *M. tuberculosis* | not defined | Hamburg |
| 10707/07 | *M. tuberculosis* | not defined | Hamburg |
| 11857/07 | *M. tuberculosis* | not defined | Hamburg |
| 11858/07 | *M. tuberculosis* | not defined | not defined |
| 1398/07 | *M. tuberculosis* | not defined | not defined |
| 1482/07 | *M. tuberculosis* | not defined | Hamburg |
| 2110/07 | *M. tuberculosis* | not defined | Hamburg |
| 2298/07 | *M. tuberculosis* | not defined | Hamburg |
| 2464/07 | *M. tuberculosis* | not defined | not defined |
| 2845/07 | *M. tuberculosis* | not defined | Hamburg |
| 3434/07 | *M. tuberculosis* | not defined | Hamburg |
| 3561/07 | *M. tuberculosis* | not defined | Hamburg |
| 3604/07 | *M. tuberculosis* | not defined | not defined |
| 3717/07 | *M. tuberculosis* | not defined | not defined |
| 4080/07 | *M. tuberculosis* | not defined | not defined |
| 4220/07 | *M. tuberculosis* | not defined | Hamburg |
| 4300/07 | *M. tuberculosis* | not defined | Hamburg |
| 4526/07 | *M. tuberculosis* | not defined | not defined |
| 4543/07 | *M. tuberculosis* | not defined | not defined |
| 4903/07 | *M. tuberculosis* | not defined | Hamburg |
| 4904/07 | *M. tuberculosis* | not defined | not defined |
| 5249/07 | *M. tuberculosis* | not defined | Hamburg |
| 6689/07 | *M. tuberculosis* | not defined | Hamburg |
| 7334/07 | *M. tuberculosis* | not defined | not defined |
| 7969/07 | *M. tuberculosis* | not defined | not defined |
| 8038/07 | *M. tuberculosis* | not defined | not defined |
| 8039/07 | *M. tuberculosis* | not defined | Hamburg |
| 8480/07 | *M. tuberculosis* | not defined | not defined |
| 8918/07 | *M. tuberculosis* | not defined | TUR |
| 9293/07 | *M. tuberculosis* | not defined | Hamburg |
| 9296/07 | *M. tuberculosis* | not defined | Hamburg |
| 9750/07 | *M. tuberculosis* | not defined | not defined |
| 987/07 | *M. tuberculosis* | not defined | not defined |
| 5494/07 | *M. tuberculosis* | S-type | S-type |
| 6753/07 | *M. tuberculosis* | S-type | S-type |
| 10126/07 | *M. tuberculosis* | S-type | S-type |
| 1979/07 | *M. tuberculosis* | TUR | TUR |
| 8919/07 | *M. tuberculosis* | TUR | TUR |
| 9598/07 | *M. tuberculosis* | TUR | TUR |
| 2279/07 | *M. tuberculosis* | Uganda I | Uganda I |
| 2009/07 | *M. tuberculosis* | Ural | Ural |
| 2380/07 | *M. tuberculosis* | Ural | Ural |
| 961/07 | *M. tuberculosis* | Ural | Ural |

CAS: Central Asien; EAI: East African Indian; LAM: Latin American Mediterranean; TUR: Turkish
