# Supplementary material for: High Resolution Discrimination of Clinical Mycobacterium tuberculosis Complex Strains Based on Single Nucleotide Polymorphisms
Source: PLoS One. 2012 Jul 2;7(7):e39855. doi: 10.1371/journal.pone.0039855 (PMC3388094; doi:10.1371/journal.pone.0039855)
Supplement: Table S3 — SNPs detected in the reference collection. (DOCX) [file pone.0039855.s003.docx]

**Table S3. SNPs detected in the reference collection.**

| Gene | SNP Name | Mutation | NT Position | AA change | Codon | N (68) |
| --- | --- | --- | --- | --- | --- | --- |
| Rv0129c | Rv0129c_309g>A^1^ | 309g>A | 309 | - | - | 3 |
| Rv0129c | Rv0129c_472g>A^1^ | 472g>A | 472 | G158S | 158 | 3 |
| Rv0129c | Rv0129c_1008t>C | 1008t>C | 1008 | - | - | 3 |
| Rv0288 | Rv0288_25c>T^1^ | 25c>T | 25 | P9S | 9 | 1 |
| Rv0288 | Rv0288_211g>T^1^ | 211g>T | 211 | A71S | 71 | 4 |
| Rv0288 | Rv0288_241a>G | 241a>G | 241 | M81V | 81 | 1 |
| Rv0388c | Rv0388c_425a>C | 425a>C | 425 | E142A | 142 | 7 |
| Rv0407 | Rv0407_236t>C | 236t>C | 236 | F79S | 79 | 1 |
| Rv0407 | Rv0407_399t>C^1^ | 399t>C | 399 | - | - | 2 |
| Rv0407 | Rv0407_409g>C^1^ | 409g>C | 409 | G137R | 137 | 2 |
| Rv0407 | Rv0407_414a>G^1^ | 414a>G | 414 | - | - | 2 |
| Rv0407 | Rv0407_430a>C^1^ | 430a>C | 430 | S144R | 144 | 2 |
| Rv0407 | Rv0407_528c>T | 528c>T | 528 | - | - | 2 |
| Rv0407 | Rv0407_624g>A | 624g>A | 624 | M208I | 208 | 1 |
| Rv0407 | Rv0407_702c>T | 702c>T | 702 | - | - | 1 |
| Rv0407 | Rv0407_809a>T^1^ | 809a>T | 809 | K270M | 270 | 3 |
| Rv0407 | Rv0407_813c>T | 813c>T | 813 | - | - | 2 |
| Rv0407 | Rv0407_886a>G^1^ | 886a>G | 886 | K296E | 296 | 4 |
| Rv0407 | Rv0407_960t>C^1^ | 960t>C | 960 | - | - | 34 |
| Rv0410c | Rv0410c_9a>G | 9a>G | 9 | - | - | 1 |
| Rv0410c | Rv0410c_108c>T | 108c>T | 108 | - | - | 1 |
| Rv0410c | Rv0410c_174a>C^1^ | 174a>C | 174 | Q58H | 58 | 1 |
| Rv0410c | Rv0410c_207g>A^1^ | 207g>A | 207 | - | - | 7 |
| Rv0410c | Rv0410c_396c>A | 396c>A | 396 | - | - | 2 |
| Rv0410c | Rv0410c_433g>C^1^ | 433g>C | 433 | D145H | 145 | 2 |
| Rv0410c | Rv0410c_475c>T^1^ | 475c>T | 475 | H159Y | - | 1 |
| Rv0410c | Rv0410c_507c>T | 507c>T | 507 | - | - | 1 |
| Rv0410c | Rv0410c_775g>C | 775g>C | 775 | E259Q | 259 | 1 |
| Rv0410c | Rv0410c_814t>C | 814t>C | 814 | - | - | 2 |
| Rv0410c | Rv0410c_1374c>T | 1374c>T | 1374 | - | - | 2 |
| Rv0410c | Rv0410c_1503a>G | 1503a>G | 1503 | - | - | 1 |
| Rv0410c | Rv0410c_1518a>G | 1518a>G | 1518 | - | - | 1 |
| Rv0410c | Rv0410c_1530c>T | 1530c>T | 1530 | - | - | 1 |
| Rv0410c | Rv0410c_1596t>C | 1596t>C | 1596 | - | - | 1 |
| Rv0410c | Rv0410c_1670a>G | 1670a>G | 1670 | K557R | 557 | 1 |
| Rv0410c | Rv0410c_1671a>G | 1671a>G | 1671 | K557R | 557 | 1 |
| Rv0410c | Rv0410c_1692t>C | 1692t>C | 1692 | - | - | 1 |
| Rv0410c | Rv0410c_1725c>G | 1725c>G | 1725 | - | - | 1 |
| Rv0410c | Rv0410c_1737c>G | 1737c>G | 1737 | - | - | 1 |
| Rv0410c | Rv0410c_1739c>T | 1739c>T | 1739 | A580V | 580 | 1 |
| Rv0410c | Rv0410c_1842g>A^1^ | 1842g>A | 1842 | - | - | 3 |
| Rv0410c | Rv0410c_1993g>A^1^ | 1993g>A | 1993 | A665T | 665 | 6 |
| Rv0410c | Rv0410c_2121g>A | 2121g>A | 2121 | - | - | 1 |
| Rv0410c | Rv0410c_2148t>C | 2148t>C | 2148 | - | - | 1 |
| Rv0410c | Rv0410c_2190t>C | 2190t>C | 2190 | - | - | 2 |
| Rv0410c | Rv0410c_2207c>A^1^ | 2207c>A | 2207 | T736K | 736 | 4 |
| Rv0557 | Rv0557_15c>T | 15c>T | 15 | - | - | 1 |
| Rv0557 | Rv0557_221c>T | 221c>T | 221 | T74M | 74 | 6 |
| Rv0557 | Rv0557_259g>A | 259g>A | 259 | A87T | 87 | 2 |
| Rv0557 | Rv0557_321t>C | 321t>C | 321 | - | - | 34 |
| Rv0557 | Rv0557_455g>C | 455g>C | 455 | R152P | 152 | 3 |
| Rv0557 | Rv0557_457c>G | 457c>G | 457 | L153V | 153 | 3 |
| Rv0557 | Rv0557_532c>G | 532c>G | 532 | R178G | 178 | 3 |
| Rv0557 | Rv0557_810c>T | 810c>T | 810 | - | - | 3 |
| Rv0557 | Rv0557_911g>A | 911g>A | 911 | G304D | 304 | 6 |
| Rv0557 | Rv0557_945c>T | 945c>T | 945 | - | - | 1 |
| Rv0557 | Rv0557_1050g>A | 1050g>A | 1050 | - | - | 3 |
| Rv0557 | Rv0557_1066c>T | 1066c>T | 1066 | P356S | 356 | 3 |
| Rv1009 | Rv1009_115a>G | 115a>G | 115 | T39A | 39 | 1 |
| Rv1009 | Rv1009_136a>G^1^ | 136a>G | 136 | I46V | 46 | 1 |
| Rv1009 | Rv1009_735c>T^1^ | 735c>T | 735 | - | - | 5 |
| Rv1009 | Rv1009_825g>C^1^ | 825g>C | 825 | E275D | 275 | 3 |
| Rv1009 | Rv1009_845g>A^1^ | 845g>A | 845 | G282E | 282 | 7 |
| Rv1009 | Rv1009_1034t>A^1^ | 1034t>A | 1034 | L345Q | 345 | 3 |
| Rv1009 | Rv1009_1038t>C^1^ | 1038t>C | 1038 | - | - | 3 |
| Rv1009 | Rv1009_1070c>T^1^ | 1070c>T | 1070 | A357V | 357 | 4 |
| Rv1009 | Rv1009_1075g>A | 1075g>A | 1075 | A359T | 359 | 3 |
| Rv1617 | Rv1617_660g>A^2^ | 660g>A | 660 | - | - | 1 |
| Rv1617 | Rv1617_660g>T^2^ | 660g>T | 660 | E220D | 220 | 21 |
| Rv1811 | Rv1811_12g>A | 12g>A | 12 | - | - | 3 |
| Rv1811 | Rv1811_45c>T | 45c>T | 45 | - | - | 3 |
| Rv1811 | Rv1811_240 c>T | 240c>T | 240 | - | - | 3 |
| Rv1811 | Rv1811_284 c>T | 284c>T | 284 | T95M | 95 | 3 |
| Rv1811 | Rv1811_320 c>t | 320c>t | 320 | A107V | 107 | 4 |
| Rv1811 | Rv1811_339c>T | 339c>T | 339 | - | - | 1 |
| Rv1811 | Rv1811_545 g>A | 545g>A | 545 | R182H | 182 | 3 |
| Rv1884c | Rv1884c_4c>T^1^ | 4c>T | 4 | H2Y | 2 | 3 |
| Rv1884c | Rv1884c_47a>G^1^ | 47a>G | 47 | H16R | 16 | 2 |
| Rv1884c | Rv1884c_473t>G | 473t>G | 473 | I158S | 158 | 1 |
| Rv1908c | Rv1908c_1388g>T | 1388g>T | 1388 | R463L | 463 | 34 |
| Rv1980c | Rv1980c_128 t>A^1^ | 128t>A | 128 | I43N | 43 | 6 |
| Rv1980c | Rv1980c_134t>C^1^ | 134t>C | 134 | M45T | 45 | 3 |
| Rv2032 | Rv2032_39c>G | 39c>G | 39 | S13R | 13 | 1 |
| Rv2032 | Rv2032_100g>T^1^ | 100g>T | 100 | A34S | 34 | 3 |
| Rv2032 | Rv2032_177g>A | 177g>A | 177 | - | - | 1 |
| Rv2032 | Rv2032_186g>C^1^ | 186g>C | 186 | - | - | 3 |
| Rv2032 | Rv2032_252a>G | 252a>G | 252 | - | - | 1 |
| Rv2032 | Rv2032_323a>T | 323a>T | 323 | E108V | 108 | 1 |
| Rv2032 | Rv2032_421c>T | 421c>T | 421 | R141C | 141 | 1 |
| Rv2032 | Rv2032_451a>C | 451a>C | 451 | I151L | 151 | 1 |
| Rv2032 | Rv2032_536a>G^1^ | 536a>G | 536 | Y179C | 179 | 1 |
| Rv2032 | Rv2032_813g>C^1^ | 813g>C | 813 | - | - | 2 |
| Rv2032 | Rv2032_837c>T | 837c>T | 837 | - | - | 1 |
| Rv2032 | Rv2032_891c>G | 891c>G | 891 | - | - | 1 |
| Rv2032 | Rv2032_915g>T | 915g>T | 915 | - | - | 1 |
| Rv2032 | Rv2032_919a>G | 919a>G | 919 | M307V | 307 | 1 |
| Rv2032 | Rv2032_953c>T^1^ | 953c>T | 953 | P318L | 318 | 7 |
| Rv2389c | Rv2389c_342c>A | 342c>A | 342 | - | - | 1 |
| Rv2428 | Rv2428_-88g>A | -88g>A | -88 | Gat>Aat | -88 | 3 |
| Rv2428 | Rv2428_18t>C | 18t>C | 18 | - | - | 1 |
| Rv2430c | Rv2430c_86g>C | 86g>C | 86 | R29T | 29 | 1 |
| Rv2430c | Rv2430c_142a>C | 142a>C | 142 | T48P | 48 | 1 |
| Rv2430c | Rv2430c_177a>T | 177a>T | 177 | - | - | 2 |
| Rv2431c | Rv2431c_3g>C | 3g>C | 3 | M1I | 1 | 1 |
| Rv2431c | Rv2431c_119g>C | 119g>C | 119 | R40P | 40 | 1 |
| Rv2450c | Rv2450c_23t>C | 23t>C | 23 | L8P | 8 | 1 |
| Rv2450c | Rv2450c_49t>C^1^ | 49t>C | 49 | - | - | 6 |
| Rv2450c | Rv2450c_59c>G^1^ | 59c>G | 59 | T20R | 20 | 3 |
| Rv2450c | Rv2450c_269c>T | 269c>T | 269 | A90V | 90 | 1 |
| Rv2450c | Rv2450c_315g>A^1^ | 315g>A | 315 | - | - | 3 |
| Rv2450c | Rv2450c_343a>G^1^ | 343a>G | 343 | I115V | 115 | 1 |
| Rv2450c | Rv2450c_417g>A^1^ | 417g>A | 417 | - | - | 3 |
| Rv2609c | Rv2609c_296t>C | 296t>C | 296 | M99T | 99 | 1 |
| Rv2609c | Rv2609c_922c>G | 922c>G | 922 | Q308E | 308 | 3 |
| Rv2610c | Rv2610c_128c>G | 128c>G | 128 | P43R | 43 | 1 |
| Rv2610c | Rv2610c_736c>A | 736c>A | 736 | R246S | 246 | 1 |
| Rv2611c | Rv2611c_247t>C | 247t>C | 247 | C83R | 83 | 3 |
| Rv2611c | Rv2611c_306a>G | 306a>G | 306 | I102M | 102 | 67 |
| Rv2611c | Rv2611c_386t>G | 386t>G | 386 | L129R | 129 | 3 |
| Rv2611c | Rv2611c_590c>G | 590c>G | 590 | S197C | 197 | 67 |
| Rv2611c | Rv2611c_630c>G | 630c>G | 630 | - | - | 1 |
| Rv2611c | Rv2611c_786t>C | 786t>C | 786 | - | - | 4 |
| Rv2612c | Rv2612c_5g>A | 5g>A | 5 | S2N | 2 | 3 |
| Rv2612c | Rv2612c_51c>T | 51c>T | 51 | - | - | 3 |
| Rv2612c | Rv2612c_152t>C | 152t>C | 152 | M51T | 51 | 3 |
| Rv2613c | Rv2613c_18c>T | 18c>T | 18 | - | - | 3 |
| Rv2613c | Rv2613c_106a>C | 106a>C | 106 | M36L | 36 | 1 |
| Rv2613c | Rv2613c_267c>T | 267c>T | 267 | - | - | 3 |
| Rv2613c | Rv2613c_349t>G | 349t>G | 349 | F117V | 117 | 1 |
| Rv2628 | Rv2628_78a>C^1^ | 78a>C | 78 | - | - | 3 |
| Rv2628 | Rv2628_79g>A | 79g>A | 79 | G27S | 27 | 1 |
| Rv2628 | Rv2628_145t>G^1^ | 145t>G | 145 | S49A | 49 | 3 |
| Rv2628 | Rv2628_176c>T^1^ | 176c>T | 176 | S59L | 59 | 28 |
| Rv2628 | Rv2628_234t>C | 234t>C | 234 | - | - | 1 |
| Rv2628 | Rv2628_248t>G^1^ | 248t>G | 248 | L83W | 83 | 5 |
| Rv2628 | Rv2628_253a>G^1^ | 253a>G | 253 | T85A | 85 | 3 |
| Rv2628 | Rv2628_273t>C^1^ | 273t>C | 273 | - |  | 2 |
| Rv2628 | Rv2628_286g>A^1^ | 286g>A | 286 | A96T | 96 | 6 |
| Rv2629 | Rv2629_191a>C | 191a>C | 191 | D64A | 64 | 3 |
| Rv2629 | Rv2629_238g>C | 238g>C | 238 | G80R | 80 | 1 |
| Rv2629 | Rv2629_352a>T | 352a>T | 352 | T118S | 118 | 2 |
| Rv2629 | Rv2629_378c>T | 378c>T | 378 | - | - | 1 |
| Rv2629 | Rv2629_534c>T | 534c>T | 534 | - | - | 2 |
| Rv2629 | Rv2629_632c>T | 632c>T | 632 | T211I | 211 | 1 |
| Rv2629 | Rv2629_813g>A | 813g>A | 813 | - | - | 1 |
| Rv2629 | Rv2629_965c>T | 965c>T | 965 | P322L | 322 | 3 |
| Rv3547 | Rv3547_67c>A^1^ | 67c>A, | 67 | - | - | 2 |
| Rv3547 | Rv3547_67c>T | 67c>T | 67 | R23W | 23 | 1 |
| Rv3547 | Rv3547_144g>T^1^ | 144g>T | 144 | - | - | 6 |
| Rv3547 | Rv3547_207c>T^1^ | 207c>T | 207 | - | - | 3 |
| Rv3547 | Rv3547_249 g>C | 249 g>C | 249 | E83D | 83 | 1 |
| Rv3547 | Rv3547_268c>G^1^ | 268c>G | 268 | L90V | 90 | 5 |
| Rv3547 | Rv3547_331g>A | 331g>A | 331 | A111T | 111 | 2 |
| Rv3547 | Rv3547_337g>A^1^ | 337g>A | 337 | D113N | 113 | 6 |
| Rv3547 | Rv3547_444t>C^1^ | 444t>C | 444 | - | - | 3 |

Hershberg et al.^1^ Keating et al. ^2^;

^1^Hershberg R, Lipatov M, Small PM, Sheffer H, Niemann S, et al. (2008) High functional diversity in Mycobacterium tuberculosis driven by genetic drift and human demography. PLoS Biol 6: e311. doi:10.1371/journal.pbio.0060311.

^2^Keating LA, Wheeler PR, Mansoor H, Inwald JK, Dale J, et al. (2005) The pyruvate requirement of some members of the Mycobacterium tuberculosis complex is due to an inactive pyruvate kinase: implications for in vivo growth. Molecular Microbiology 56: 163–174. doi:10.1111/j.1365-2958.2005.04524.x.
