# Supplementary material for: High Resolution Discrimination of Clinical Mycobacterium tuberculosis Complex Strains Based on Single Nucleotide Polymorphisms
Source: PLoS One. 2012 Jul 2;7(7):e39855. doi: 10.1371/journal.pone.0039855 (PMC3388094; doi:10.1371/journal.pone.0039855)
Supplement: Table S4 — SNPs detected in Hamburg collection 2007. (DOCX) [file pone.0039855.s004.docx]

**Table S4. SNPs detected in Hamburg collection 2007.**

| Gene | SNP Name | Mutation | NT Position | AA change | Codon |
| --- | --- | --- | --- | --- | --- |
| Rv0129c | Rv0129c_309g>A^1,3^ | 309g>A | 309 | - | - |
| Rv0129c | Rv0129c_472g>A^1,3^ | 472g>A | 472 | G158S | 158 |
| Rv0410c | Rv0410c_9a>G | 9a>G | 9 | - | - |
| Rv0410c | Rv0410c_207g>A^2,3^ | 207g>A | 207 | - | - |
| Rv0410c | Rv0410c_299c>T | 299c>T | 299 | P100L | 100 |
| Rv0410c | Rv0410c_433g>C^3^ | 433g>C | 433 | D145H | 145 |
| Rv0410c | Rv0410c_475c>T^3^ | 475c>T | 475 | H159Y | - |
| Rv0410c | Rv0410c_1842g>A^1,3^ | 1842g>A | 1842 | - | - |
| Rv0410c | Rv0410c_1892c>T | 1892c>T | 1892 | P631L | 631 |
| Rv0410c | Rv0410c_1993g>A^1,3^ | 1993g>A | 1993 | A665T | 665 |
| Rv0410c | Rv0410c_2117t>C^1,3^ | 2117t>C | 2117 | F706S | 706 |
| Rv0557 | Rv0557_68c>T | 68c>T | 68 | S23L | 23 |
| Rv0557 | Rv0557_221c>T^1^ | 221c>T | 221 | T74M | 74 |
| Rv0557 | Rv0557_321t>C^2^ | 321t>C | 321 | - | - |
| Rv0557 | Rv0557_455g>C^1^ | 455g>C | 455 | R152P | 152 |
| Rv0557 | Rv0557_457c>G^1^ | 457c>G | 457 | L153V | 153 |
| Rv0557 | Rv0557_532c>G^1^ | 532c>G | 532 | R178G | 178 |
| Rv0557 | Rv0557_801g>A | 801g>A | 801 | - | - |
| Rv0557 | Rv0557_810c>T^1^ | 810c>T | 810 | - | - |
| Rv0557 | Rv0557_829c>A | 829c>A | 829 | H277N | 277 |
| Rv0557 | Rv0557_890c>T | 890c>T | 890 | P297L | 297 |
| Rv0557 | Rv0557_911g>A^1^ | 911g>A | 911 | G304D | 304 |
| Rv0557 | Rv0557_980g>T | 980g>T | 980 | R327L | 327 |
| Rv1009 | Rv1009_575t>C | 575t>C | 575 | V192A | 192 |
| Rv1009 | Rv1009_724g>A^1,3^ | 724g>A | 724 | E242K | 242 |
| Rv1009 | Rv1009_793g>A^3^ | 793g>A | 793 | V265M | 265 |
| Rv1009 | Rv1009_845g>A^2.3^ | 845g>A | 845 | G282E | 282 |
| Rv1009 | Rv1009_872g>A | 872g>A | 872 | C291Y | 291 |
| Rv1009 | Rv1009_996c>T | 996c>T | 996 | - | - |
| Rv1009 | Rv1009_1070c>T^1,3^ | 1070c>T | 1070 | A357V | 357 |
| Rv1009 | Rv1009_1075g>A^1^ | 1075g>A | 1075 | A359T | 359 |
| Rv1811 | Rv1811_12g>A^1^ | 12g>A | 12 | - | - |
| Rv1811 | Rv1811_48a>C | 48a>C | 48 | - | - |
| Rv1811 | Rv1811_132c>T | 132c>T | 132 | - | - |
| Rv1811 | Rv1811_240c>T^1^ | 240c>T | 240 | - | - |
| Rv1811 | Rv1811_284c>T^1^ | 284c>T | 284 | T95M | 95 |
| Rv1811 | Rv1811_339c>T | 339c>T | 339 | - | - |
| Rv1811 | Rv1811_458t>G | 458t>G | 458 | V153G | 153 |
| Rv1811 | Rv1811_545g>A^1^ | 545g>A | 545 | R182H | 182 |
| Rv1811 | Rv1811_697g>C | 697g>C | 697 | A233P | 233 |
| Rv1908c | Rv1908c_1388g>T^2^ | 1388g>T | 1388 | R463L | 463 |
| Rv2450c | Rv2450c_20c>T | 20c>T | 20 | T7M | 7 |
| Rv2450c | Rv2450c_49t>C^1,3^ | 49t>C | 49 | - | - |
| Rv2450c | Rv2450c_59c>G^1,3^ | 59c>G | 59 | T20R | 20 |
| Rv2450c | Rv2450c_269c>T | 269c>T | 269 | A90V | 90 |
| Rv2450c | Rv2450c_322g>T | 322g>T | 322 | E108* | 108 |
| Rv2450c | Rv2450c_343a>G^3^ | 343a>G | 343 | I115V | 115 |
| Rv2613c | Rv2613c_18c>T^1^ | 18c>T | 18 | - | - |
| Rv2613c | Rv2613c_429g>C | 429g>C | 429 | - | - |
| Rv2628 | Rv2628_4t>C^1,3^ | 4t>C | 4 | S2P | 2 |
| Rv2628 | Rv2628_47c>T^3^ | 47c>T | 47 | P16L | 16 |
| Rv2628 | Rv2628_81c>T | 81c>T | 81 | - | - |
| Rv2628 | Rv2628_176c>T^2,3^ | 176c>T | 176 | S59L | 59 |
| Rv2628 | Rv2628_286g>A^1.3^ | 286g>A | 286 | A96T | 96 |
| Rv2629 | Rv2629_48c>T | 48c>T | 48 | - | - |
| Rv2629 | Rv2629_191a>C^1^ | 191a>C | 191 | D64A | 64 |
| Rv2629 | Rv2629_327g>A | 327g>A | 327 | - | - |
| Rv2629 | Rv2629_965c>T^1^ | 965c>T | 965 | P322L | 322 |
| Rv3547 | Rv3547_144g>T^1,3^ | 144g>T | 144 | - | - |
| Rv3547 | Rv3547_200t>C | 200t>C | 200 | L67P | 67 |
| Rv3547 | Rv3547_337g>A^1,3^ | 337g>A | 337 | D113N | 113 |

^1^genotype specific SNPs; ^2^lineage specific SNPs;* stop codon; ^3^ Hershberg et al. [7]
