# Supplementary material for: High Resolution Discrimination of Clinical Mycobacterium tuberculosis Complex Strains Based on Single Nucleotide Polymorphisms
Source: PLoS One. 2012 Jul 2;7(7):e39855. doi: 10.1371/journal.pone.0039855 (PMC3388094; doi:10.1371/journal.pone.0039855)
Supplement: Table S5 — Additional variations in strains of the reference collection. (DOCX) [file pone.0039855.s005.docx]

**Table S5. Additional variations in strains of the reference collection.**

| Sample Name | Species | Genotype | Gene | Mutation | NT Position | Nucleotides |
| --- | --- | --- | --- | --- | --- | --- |
| 416/01  8753/00  1479/99  7011/02  7739/01 | *M. microti*  *M. microti*  *M. microti*  *M. pinipedii*  *M. pinipedii* | Llama  Llama  Vole  Seal  Seal | Rv0388c | deletion | 438 | t |
| 3040/99 | *M. canettii* | Canettii | Rv2389c | deletion | 1-144 | atgacaccgggtttgcttactactgcgggtgctggccgaccacgtgacaggtgcgccaggatcgtatgcacggtgttcatcgaaaccgccgttgtcgcgaccatgtttgtcgcgttgttgggtctgtccaccatcagctcgaaa |
| 9915/01 | *M. tuberculosis* | Dehli/CAS | Rv2428 | deletion | -75 | t |
| 8885/03 | *M. tuberculosis* | LAM | Rv2430c | deletion | 6-117 | tttcgaagcgtacccaccggaggtcaactccgccaacatatatgccggccccggtcctgactcgatgttggctgccgccagggcgtggaggtcgttggatgtggaaatgacg |
| 2679/03 | *M. tuberculosis* | Ural | Rv2450c | deletion | 491 – 519 (stop codon) | cctggccggtctgcggccgccgcggctga |
| 3041/99  3151/08 | *M. canettii* | Canettii | Rv2628 | insertion | 91 | g |
