# Supplementary material for: High Resolution Discrimination of Clinical Mycobacterium tuberculosis Complex Strains Based on Single Nucleotide Polymorphisms
Source: PLoS One. 2012 Jul 2;7(7):e39855. doi: 10.1371/journal.pone.0039855 (PMC3388094; doi:10.1371/journal.pone.0039855)
Supplement: Table S6 — Additional variations in strains from Hamburg, Germany. (DOCX) [file pone.0039855.s006.docx]

**Table S6. Additional variations in strains from Hamburg, Germany.**

| Sample Name | Species | Genotype | Gene | Mutation | NT Position | Nucleotides |
| --- | --- | --- | --- | --- | --- | --- |
| \| 1979/07 \| \| --- \| \| 8919/07 \| \| 9598/07 \| | *M. tuberculosis*  *M. tuberculosis*  *M. tuberculosis* | TUR  TUR  TUR | Rv2450c | deletion | 475 – 576 | tcgcagggtatccgcgcctggccggtctgcggccgccgcggctgaccaccgcgaaaagacgcaaaagctcccaaatccggtcggatttgggagcttttgcgt |
| 10583/07  11857/07  1482/07  2298/07  3434/07  4300/07  4903/07  5249/07  6689/07  9296/07 | *M. tuberculosis*  *M. tuberculosis*  *M. tuberculosis*  *M. tuberculosis*  *M. tuberculosis*  *M. tuberculosis*  *M. tuberculosis*  *M. tuberculosis*  *M. tuberculosis*  *M. tuberculosis* | Hamburg  Hamburg  Hamburg  Hamburg  Hamburg  Hamburg  Hamburg  Hamburg  Hamburg  Hamburg | Rv2450c | deletion | 506 – 575 | gccgccgcggctgaccaccgcgaaaagacgcaaaagctcccaaatccggtcggatttgggagcttttgcg |
